# Workplace Interventions to Reduce Occupational Stress for Older Workers: A Systematic Review

**DOI:** 10.3390/ijerph19159202

**Published:** 2022-07-27

**Authors:** Daniel Subel, David Blane, Jessica Sheringham

**Affiliations:** 1 Institute of Epidemiology and Health Care, University College London, London WC1E 6BT, UK; 2 Faculty of Medicine, School of Public Health, Imperial College London, London SW7 2AZ, UK; d.blane@imperial.ac.uk; 3 Department of Applied Health Research, University College London, London WC1E 6BT, UK; j.sheringham@ucl.ac.uk

**Keywords:** intervention, workplace, occupational stress, older workers

## Abstract

The working life of individuals is now longer because of increases to state pension age in the United Kingdom. Older workers may be at particular risk in the workplace, compared with younger workers. Successful workplace interventions to reduce occupational stress amongst older workers are essential, but little is known about their effectiveness. The aim is to evaluate current evidence of the effectiveness of interventions for reducing stress in older workers in non-healthcare settings. Four database searches were conducted. The search terms included synonyms of “intervention”, “workplace” and “occupational stress” to identify original studies published since 2011. Dual screening was conducted on the sample to identify studies which met the inclusion criteria. The RoB 2.0 tool for RCTs was used to assess the risk of bias. From 3708 papers retrieved, ten eligible papers were identified. Seven of the papers’ interventions were deemed effective in reducing workplace stress. The sample size for most studies was small, and the effectiveness of interventions were more likely to be reported when studies used self-report measures, rather than biological measures. This review indicates that workplace interventions might be effective for reducing stress in older workers. However, there remains an absence of high-quality evidence in this field.

## 1. Introduction

By 2040, it is predicted that one in every two people of working age will be aged 65 or over [1]. The global ageing population has resulted in government concerns regarding the future of the workplace [2]. The increase in life expectancy and the lack of equitable social resources available has been a catalyst for most European governments to increase the state pension age [3].

Prolonging the working life of individuals cannot be done without due diligence and needs to be medically supervised, as suggested by MISPA (Mitigating Increases in the State Pension Age) [4]. Before governments can continue increasing state pension age, it needs to be assessed how this can be conducted, without damaging or harming the health of workers affected by these changes–particularly workers in physically demanding and highly stressful occupations.

Older workers face greater or different hazards to their health than younger workers. Bravo et al.’s [5] review found that in 50% of the papers they reviewed, older workers were at a much greater risk of fatal workplace injuries, when compared to their younger counterparts. Older workers are more likely to have pre-existing long-term health conditions, which can affect their capacity to work or the kinds of work they are able to sustain. There is also evidence that they experience greater sickness absence [6].

Stress—adverse reactions to excessive pressure—and burnout are recognised as a major risk to the health of all workers [7,8]. There is conflicting evidence whether older workers are at a greater risk of stress than younger workers [9]. It is clear, however, that older workers are likely to face different stressors to younger workers, not just through pressures within the workplace, but also through additional caring responsibilities outside of work [4]. Moreover, there is agreement that, despite legislation to prevent it, there is evidence that older workers are subject to age discrimination [10]. Therefore, interventions to improve workplace health in older workers may well need to be different to those of younger workers because it cannot be assumed that the problems they face, or the mechanisms by which interventions work, will be the same.

Based on currently available research, very little is known about this topic. Evidence of the effectiveness of workplace interventions for older workers is lacking. Poscia et al.’s [3] systematic review found a paucity of high-quality evidence on workplace health promotion for older workers. There was a suggestion that active workplace interventions help improve the health of older workers, but included studies used small, convenience samples not representative of the working population.

Pieper et al.’s [11] more recent review of reviews on workplace health promotion interventions found that psychological interventions, such as stress management, cognitive behavioural therapy and mindfulness-based interventions have the ability to significantly reduce stress. However, Pieper et al. found few reviews specifically focusing on older workers and they reported that there was insufficient evidence to conclude that psychological interventions were the most successful and effective to reduce occupational stress amongst older workers. Interventions were predominately targeted towards white-collar workers, teachers, and healthcare providers. Interventions for healthcare providers may be of limited generalisability to other settings, given the specific nature of healthcare settings and healthcare work and the hazards that may present in this environment. When assessing previous studies on this topic, the overwhelming majority focus upon younger people employed in advantaged occupations, using small cohort sizes. Furthermore, they use inconsistent and haphazard outcome measures to assess interventions’ successes, which results in studies being unrepresentative, difficult to replicate and unable to demonstrate the impact on increased state pension age for older workers.

Before policy makers can enact changes to state pension age, they must have access to a sufficient level of high-quality research which has outlined the impact on individuals working longer, as well as interventions used to retain older workers. This information must also be accessible to employers, so they are made aware of the most successful interventions in the workplace to reduce occupational stress and maintain their workforce. This article intends to provide policy makers and employers a review of the current literature and research in this field. This study also has the potential to provide union representatives, and workers themselves, with evidence for them to vouch to their employers for adequate, appropriate, and successful interventions in their workplaces.

This systematic review sought to answer the question:

“What is the evidence of effectiveness of workplace interventions for reducing occupational stress in older workers outside of the healthcare sector?”

The objectives were to:

(1) Identify and appraise papers evaluating the effectiveness of workplace stress reduction interventions on older workers.
(2) Describe the types of interventions and measures of effectiveness used
(3) Summarise the evidence of effectiveness of interventions
(4) Identify existing knowledge gaps in the literature which require further research

## 2. Materials and Methods

### 2.1. Search Strategy

PRISMA guidelines for reporting systematic reviews were followed throughout the process of this review [12], see checklist in Supplementary Materials. Four database searches were conducted: OvidMedline, PsycInfo, Scopus and Web of Science. After an initial literature search, a PICO model was developed (see Table 1), which helped form the database search terms for the review [13]. Previously systematic reviews’ search terms, including Pieper et al. [11] and Poscia et al. [3], helped to inform the search terms. The search term combinations were first applied in OvidMedline, which uses MeSH terms, and then modified and adapted for use in the other databases (see Table 2). In all the databases, the presence of key words was sought in “all fields”, which would detect the terms in key words, titles, abstracts and full papers. Initially age terms were included in the search strategy, but this resulted in an improbably low number of results retrieved, so this term was dropped. The searches took place throughout the first week of August 2021, therefore only research published before 31 July 2021 were included in this review.

### 2.2. Inclusion/Exclusion Criteria

Table 3 depicts the inclusion/exclusion criteria. Eligible papers had to report an intervention in a non-health sector workplace, specifically focusing on older workers. The papers had to have been conducted in an Organisation for Economic Co-operation and Development (OECD) country, to ensure findings have some relevance to the United Kingdom (UK) context [14]. Studies without a control group or baseline data, or without an aim of reducing workplace stress, were excluded. The authors did not set out to select papers which specified a specific control condition but sought papers which described what interventions were compared with. Qualitative papers, such as focus groups or interviews, were excluded from this review as quantitative papers were deemed to illustrate more objective results and are more likely to be conducted on a large number of participants.

The definition of an older worker was developed by adapting multiple definitions from various sources. Firstly, if the paper classified the intervention or participants as older workers, regardless of the mean age, these interventions were deemed to be focused on older workers. Secondly, for OECD countries, the average age at which an individual reached normal pension age in 2016 was 63.7 years old for women and 64.3 years old for men [15]. If the mean age of participants in a paper were within 15 years of normal pension age, it was concluded that older workers were included in this intervention.

### 2.3. Study Selection and Screening

Papers from the four databases were exported to Microsoft Excel. Title and abstracts of all papers screened by DS (author and reviewer) and a secondary reviewer (AH). Any papers which were unclear or resulted in polarized views, were then resolved by discussion with a third reviewer and co-author (JS). After the title and abstract screening, the remaining papers underwent a full-text screening.

Each paper that met the inclusion criteria on screening was carefully assessed for its relevance to older workers. Papers that were specifically focused on older workers were placed in the primary dataset. Papers where data on the effectiveness of the intervention on older workers was included, but without a specific focus on older workers, comprised the secondary dataset.

### 2.4. Data Extraction and Critical Appraisal

Data were extracted from all eligible papers used a data extraction form by DS, with a sample checked by JS (see Appendix A: Data Extraction Form) to cover features including: study design and employment setting; the age of participants; nature of the intervention; reported effectiveness. Interventions were coded into three categories–psychological interventions, educational interventions, and physical interventions. Outcome measurements were grouped by whether self-report or biological samples were used to measure stress.

The RoB 2.0 tool (Risk of bias in randomised trials) [16] was used by DS and JS in each paper to form a judgement about the risk of bias across six different domains. The RoB 2.0 tool was chosen as it enabled the reviewers to form their own assessment of an article’s quality, in regard to its risk of different types of biases. If a domain or the overall judgement was deemed to have a high risk of bias, this meant that the reviewers believed that there was an issue with the paper that substantially lowered their confidence in the results. Some concerns of bias indicated that a paper included an issue which could potentially lower the reviewer’s confidence in the results. If the overall judgement was that the paper had a low risk of bias, this meant that the reviewers were confident that the study results were valid.

The included studies were described, and the characteristics and methods for ascertaining stress levels were summarised. Based upon what was written in each paper, the effectiveness of the interventions was summarised, using quantitative data to assess the success of each intervention.

## 3. Results

### 3.1. Characteristics of Included Studies

**Figure 1:**
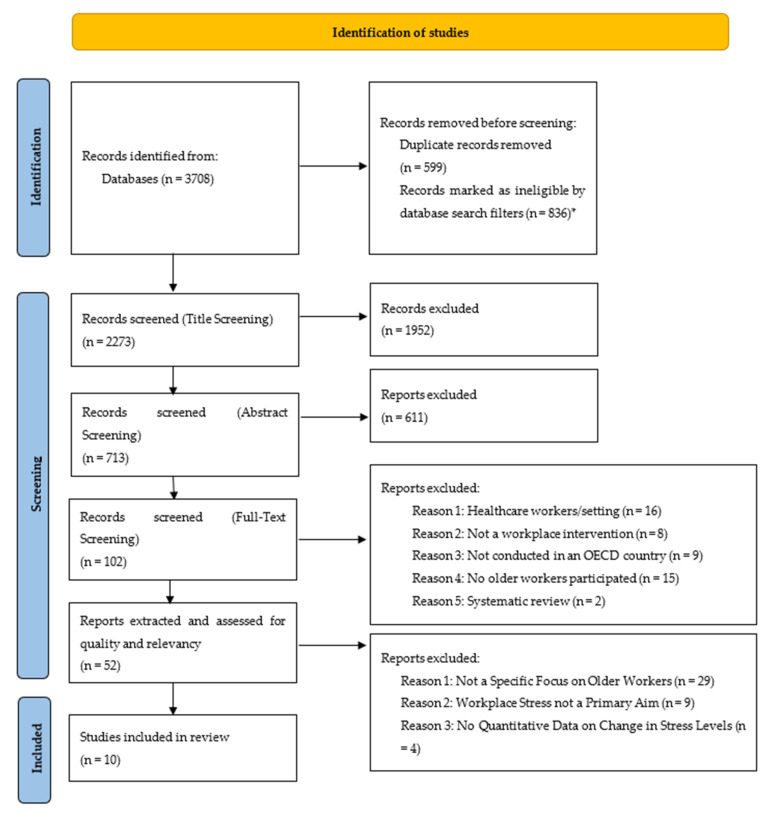
PRISMA Flow Chart (Adapted from Page et al. [12]) * Papers were automatically excluded using filters in the search databases where they were outside of our date range and language of publication.

From 3708 papers identified in the database searches, ten papers met the inclusion criteria (Figure 1). Five papers had a specific focus on older workers (the primary dataset). A further five papers did not have a specific focus of the research on older workers (the secondary dataset). As the mean age of participants in both datasets were similar (see Table 4), they are considered as one dataset in the rest of the paper.

Five papers were conducted in the United States [17,18,19,20,21]; the other five originated from Europe (Germany [22], the Netherlands [23], Finland [24], Italy [25] and Norway [26]). The number of participants ranged from 14–779, with three studies have less than 40 participants. Only one study included over 500 participants [24] (see Table 4).

**Table 4:** Description of Studies.

Study (First Author, Year)	Country	Study Design	Focus on Older Workers?	Participant’s Occupation	Age of Participants	No. of Participants (and Dropouts)
Primary Dataset						
Hughes, 2011 [17]	United States	Randomised Controlled Trial (RCT)	Yes	University Staff	51 (Mean)	423 (56 Dropouts)
Malarkey, 2013 [18]	United States	Randomised Controlled Trial	Yes	University Faculty Staff	50 (Mean)	186 (0 Dropouts)
Cook, 2015 [20]	United States	Randomised Controlled Trial	Yes	Tech Company Workers	59 (Median) * 50–68 (Range)	278 (0 Dropouts)
Fischetti, 2019 [25]	Italy	Randomised Controlled Trial	Yes	Police Officers	46.8 (Mean)	20 (0 Dropouts)
Calogiuri, 2016 [26]	Norway	Randomised Controlled Trial	Yes	Office Workers	49 (Median) * 41–47 (Range)	14 (3 Dropouts)
Secondary Dataset						
Aikens, 2014 [19]	United States	Randomised Controlled Trial	No	Chemical Company Employees	41.5 (Median) * 18–65 (Range)	89 (23 Dropouts)
Largo-Wight, 2017 [21]	United States	Randomised Controlled Trial	No	University Office Staff	48.8 (Mean)	37 (0 Dropouts)
Limm, 2011 [22]	Germany	Randomised Controlled Trial	No	Lower and Middle Level Managers at a Manufacturing Plant	40.9 (Mean) 18–65 (Range)	174 (20 Dropouts)
Hoeve, 2021 [23]	Netherlands	Quasi-Experimental	No	Police Officers	49 (Mean) 30–63 (Range)	82 (19 Dropouts)
Ojala, 2019 [24]	Finland	Non-Randomised Trial	No	Public Sector Workers *	49.9 (Mean) 21–64 (Range)	779 (217 Dropouts)

* Median has been calculated by the researcher as the midpoint between the range. In Ojala’s study, Public Sector. workers included construction and transport workers, office workers, food services and managerial specialists.

Three studies were conducted with university faculty staff [17,18,21], and three in manufacturing or technical environments [19,20,22]. Two studies were conducted amongst police officers [23,25]. The remaining two studies were conducted with office workers [24,26].

The age of participants was described in two ways (Table 4). Six studies described the age range of participants in the intervention; the upper limit for the age range was between 57 and 68; the lower limit for the age range was 18 to 50. Three papers only included participants over the age of 40, with Calogiuri et al.’s [26] paper using only participants older than 50 years old. In the seven papers that documented the mean age of participants, mean age was over 40.9 years. Five papers had a mean age of over 48 years [17,18,21,23,24].

### 3.2. Risk of Bias

None of the papers had an overall high risk of bias (Table 5). Four papers were judged to have a low risk of bias. Some bias concerns were identified in six papers. In nine out of the ten papers there was a lack of detail on the randomisation of participants, which may have led to post-test reporting bias by participants exaggerating the effects of the intervention. Most papers showed a strong adherence to the intended intervention. Fischetti et al.’s [25] study showed a potential high risk of bias in the measurement of outcome. While the study used validated scales to assess stress, the score was high because of the study’s pre-post evaluation design. It is possible that participants may be subject to bias in overestimating the effects of participation on their well-being.

### 3.3. Study Methods

The most common form of intervention was psychological interventions (*n* = 8). Psychological interventions included mindfulness-based, cognitive behavioural therapy and stress management interventions. Three studies used physical interventions, which involved exercise, walking, weight training or circuit training programmes [20,25,26]. One paper included an educational intervention [17] focused on health education (Table 6).

Of the ten papers in this review, five papers [19,20,22,24,25] reported that the control group received no intervention during the research but were waitlisted to participate in the intervention at a later date. In Malarkey et al.’s [18] study, the participants in the control group received a lifestyle and educational intervention, compared with the mindfulness-based intervention that the experimental group received. Hughes et al.’s [17] study control group received a light level of health education compared with the experimental group, who received the health promotion intervention. Hoeve et al.’s [23] control group received a regular education intervention, without any mindfulness training. Two papers’ control groups [21,26] had either an indoor standard work break or indoor exercise, compared to the experimental groups whose interventions were conducted outdoors. No conclusive pattern emerged between which control condition was in place and the outcome of the intervention. Table 6 illustrates that of the five interventions [19,20,22,24,25] in which the control group received no intervention, three of these papers reported an effective intervention in the experimental group.

Six papers conducted their interventions in the workplace offices, two papers were carried out via online means in the workplace, and a further two papers took place outside of the workplace, in green areas and nature. 

All papers in this review used self-reported questionnaires to collect data on stress. Three of these papers also collected cortisol levels, either from saliva samples or blood tests [18,22,26]. Four of the papers used the Perceived Stress Scale Questionnaire to assess the level of psychological stress perceived in participants. 

The shortest intervention took place over the course of two weeks [26]. Three of the papers’ interventions took place for over six months, including follow up time [17,22,24]. The longest duration for intervention was Hughes et al.’s [17] 12-month study.

### 3.4. Study Findings

Changes in stress levels as a result of each intervention are reported in Table 6. In seven out of the ten studies [19,21,22,23,24,25,26], there was improvement in at least one measure of self-reported stress levels. However, none of the three studies that measured cortisol levels [18,22,26] found any significant differences between the intervention and the control group’s cortisol levels. 

Three interventions [17,18,20] showed no evidence of effectiveness on any measure. There were no consistent patterns in terms of the intervention type (psychological, physical educational), workplace setting or delivery method between effective and ineffective interventions.

## 4. Discussion

### 4.1. Main Findings

The evidence of the effectiveness of interventions to reduce stress in older workers was varied. Seven out of the ten papers reported some effectiveness in reducing self-reported stress in older workers as a result of interventions. Studies that measured cortisol levels did not report any reduction in stress. Most of the interventions were psychological in nature, but there was no difference in reported effectiveness between psychological, physical, or educational interventions. It should be noted that most of these interventions were only short-term, and therefore, longer-term impacts of these interventions are not clearly demonstrated.

### 4.2. Methodological Considerations

There were some important limitations in the studies included in the review. Firstly, the number of participants reported in each study was generally low, with three papers comprising studies of less than 40 participants and only one study with more than 500. Due to the low number of participants, it is difficult to generalise the results of these interventions to the broader population [27]. In most of the studies, participants had to volunteer to take part. In some studies, it was not clear how many employees that were eligible declined to take part so the acceptability of such interventions in the workplace cannot be concluded from this study.

Secondly, none of the ten papers observed the longer-term impacts of the interventions. Whilst papers stated or implied that the interventions were longer-term solutions to the problem of occupational stress amongst older workers, they provide no conclusive evidence of long-term benefit. The concern regarding the long-term effects associated with workplace interventions has also been discussed by others. Steenstra et al. [28] reported how the effect of interventions require a very long follow-up, which is extremely difficult to achieve and maintain. They concluded that the interventions’ effect would most likely dilute over time and not result in any long-term benefits. Similarly, in this review, two out of the three papers which had interventions lasting more than 8 months were shown to have mixed or no effect on reducing workplace stress. This is suggestive evidence in support of Steenstra et al.’s conclusions that the impact of workplace interventions to reduce stress could have little to no long-term benefit if the intervention is not maintained in the workplace. It is also possible maintenance of a short-term intervention is not enough; workplaces may need different kinds of approaches to maintain reductions in stress levels in the longer term.

Thirdly, there was a range of self-report questionnaires used, which collected data on various aspects relating to stress, mental health, or other factors. When analysing the interventions, as different measurement outcomes are used, it can cause difficulties in understanding which intervention is the most effective. 

There were some limitations also in the conduct of this review. Only papers published in English were included in the review. Studies which were written and published in other languages were removed at the first stage of screening. Whilst the majority of papers which were found in the database search stage of the review were written in English, those in other languages may have been beneficial to include in this review. Using free, online translation software to translate any non-English studies has become more common in academic reviews, and if this research was to be conducted again in the future, including non-English studies, and using translation software should be strongly considered. It was also beyond the scope of the review to include qualitative studies. Whilst these would not have definitely addressed questions of effectiveness, they could have provided useful insights into why intervention achieved their effects. The RoB 2.0 tool which was used for performing the critical appraisal does not prompt consideration of wider aspects of quality and relevance, for example, what the control conditions were. This could be seen as a potential limitation in several of the papers in the review.

A further challenge faced in this review was the ambiguity regarding the definition of an older workers. The initial search terms included specific terms and synonyms for “older worker”. However, this resulted in a very small number of papers being retrieved. Therefore, this search term was removed and at abstract and full paper screening, the reviewers determined which papers focused on older workers and which did not. Eliminating “older workers” as a search term in the database search led to a potential risk that relevant literature, with a clear focus on older workers, may have been overlooked. However, “older worker” was hard to define partly because the classification of an older worker varies across countries. The ELSA (English Longitudinal Study of Ageing) and the JSTAR (Japanese Study of Ageing and Retirement) both describe workers over the age of 50 as “older workers”, however, Kingston and Jagger [29] argue that cohort studies with the lower age limit of 50 to 65 years old, often have fewer very old people in the studies, therefore, are not fully representative of older workers. The nature of the risk of being an older worker varies in the context of workplace settings, occupations and job demands. For example, as seen in Fischetti et al.’s [25] and Hoeve et al.’s [23] research, police officers may be more at risk of injury at a younger age, due to the physical nature of their occupation. This may result in police officers aged 40 being deemed as older workers in their profession, although at their chronological age, in society and in other professional groups, they would be classified as younger. However, at this age, it is possible for some police officers that the nature of their work may change, to become more ‘desk based’. In this case, the current workplace exposures may be more similar to office workers, but the prior exposures they faced from working in communities may have long lasting and distinct effects on their health that are not experienced by those who have spent their entire careers in office-based jobs. Due to the small number of studies identified, this review was not able to explore the differences in the nature of interventions across workplaces. This is needed in future because different causes of stress based upon a range of diverse types of employment may affect the sustainability and the effectiveness of interventions to reduce workplace stress.

### 4.3. Interpretation of Findings and Comparison to Previous Studies

Of the ten papers sourced for this review, only five reported a specific focus on older workers, demonstrating the lack of robust and available literature on this topic. This finding is consistent with older systematic reviews researching workplace interventions for older workers [3,11,28]. More than ten years ago, Crawford et al. [30] urged for more research to be conducted on health and safety management interventions for workers over the age of 50 in relation to the physical and psychological changes that occur when workers reach this age. 

Interventions that used self-reported measures appeared more effective when compared to biological measures. However, it is important to note that self-reports and biological samples measure different things. Taking part in an intervention may improve subjective well-being in an individual, even if it has no biological effect. This does not imply that the intervention was unsuccessful or ineffective. Indeed, McDonald [31] suggests that gaining self-reported data from participants is the most logical way to learn more about an individual. Arguably, an individual’s subjective well-being is what would keep them in the workplace. 

Whilst it is understood that older workers might not always face more workplace stress compared to younger workers, they could be more at risk of specific stressors connected to responsibilities outside of work, age discrimination and physical health conditions that are more common in older age [32]. In the ten papers’ interventions, there was not enough description regarding the extent to which specific stressors associated with older age were addressed. It is, however, significant that there were five studies that did not seek to focus on older workers, potentially overlooking distinct stressors. In these studies, it is also possible that the overall effectiveness could have been driven by higher effectiveness in younger populations but there was not sufficient data reported in these papers on effects by participant age to explore this. The context in which the interventions effect change may be important. Interventions in the workplace, which are promoted and supported by employers may encourage participants not only to take part in the intervention, but also to make changes to their lifestyle and behaviour, which, in turn, would ultimately improve their well-being and decrease their stress levels at work [33].

### 4.4. The Significance of the Review and Public Health Implications

Since Crawford et al.’s [30] review was undertaken, policy and demographic changes have lead to a higher proportion of older workers in many countries, increasing the importance of health and safety interventions for workers over the age of 50. The need for such research has not been addressed and the knowledge gaps that were present in the literature remain. 

This review has demonstrated that there is still not sufficient research available for governments and policy makers to make an informed decision on the impact of increasing state pension age on the population. If they are determined to extend the working life of individuals, governments will need to ensure that there is no detriment to the health of older workers. 

The lack of high-quality literature on this topic results in this review being unable to provide any definitive conclusions regarding the most effective and successful workplace intervention to deal with occupational stress. This systematic review can be updated to illustrate newly published literature about older workers’ well-being in years to come. The significance of implementing a successful intervention to promote and maintain the health of older workers is vital for the longer-term wealth creation and sustaining of both the economy and health of the population [34]. 

This review has not shown an adequate amount of successful workplace interventions to support older workers’ occupational stress to mitigate the public health implications of raising state pension age, as reported by MISPA [4]. More extensive and robust research is required to illustrate to both employers and policy makers that increasing state pension age will result in; increased morbidity and mortality rates for those in demanding occupations; overwhelming the already sparse healthcare services-both for occupational health and primary care; and, worsening the health for workers who are already ill. Careful considerations need to be made to ensure that older workers are not adversely harmed by increases to state pension age. It is fundamental that interventions, which have been proven successful for older workers, must be introduced into more workplaces to ensure a smoother transition for older workers who are now working longer.

## 5. Conclusions

As the population ages, and statutory pension age increase, the proportion of older workers will increase in the workplace. Older workers face distinct and sometimes greater risks to health and well-being compared with younger workers, which may place them at particular risk of stress. This review found some promising evidence that interventions in the workplace can improve self-reported stress in older workers in the short term. It also highlighted the paucity of studies with interventions specifically designed for older workers. Further studies are required to understand longer term impacts of workplace interventions on older workers and to elucidate what type of intervention is most likely to be effective in different workplace settings.

## Figures and Tables

**Table 1:** The PICO model.

PICO Term	Detail
Population	Older workers, in the Organisation for Economic Co-Operation and Development (OECD) country, in non-health sector jobs.
Intervention	All interventions occurring in the workplace, including medication, educational and exercise interventions.
Control	Comparison with control conditions as described in each of the papers in the review
Outcome	Reduced workplace stress

**Table 2:** Search Terms.

Database	Programmes Search Terms	Setting Search Terms	Outcome Search Terms	Papers per Database
OvidMedline	Intervention.mp. OR Psychosocial Intervention/	Workplace/ OR Workplace.mp.	Burnout, Professional/OR	2444
			Occupational Stress.mp. OR	
			Stress, Psychological/OR Occupational Stress/OR Occupational Diseases	
Scopus	Intervention OR Programme OR Program	Workplace OR Office OR “Work Centre”	“Occupational Stress” OR “Professional Burnout” OR “Psychological Stress”	701
Web of Science	Intervention OR Programmes OR Program	Workplace * OR Office * OR “Work Centre”	“Occupational Stress” OR “Professional Burnout” OR “Workplace Stress” OR “Job Stress” OR “Psychological Stress”	964
PsycInfo	Exp Workplace Intervention/OR Intervention.mp. OR exp intervention	Exp Workplace Intervention/OR Workplace.mp.	Occupational Stress.mp. OR exp Occupational Stress/	469
Total Papers		3708 Papers		

* MeSH terms are indicated with a “/” after the search term. Programmes, setting and outcome search terms were combined with “AND” in each database.

**Table 3:** Inclusion/Exclusion Criteria.

Order	Criteria	Inclusion Criteria	Exclusion Criteria
1	Language		Paper not published in English
2	Date of Publication	Published between 1 January 2011–31 July 2021	
3	Access to Publication	Full Paper Access via UCL/Online	Paper not fully available online
4	Type of Paper		Papers without an Abstract
5	Publication Type	Original Studies Peer Reviewed Studies	Systematic Reviews Editorials Dissertations Not Fully Published Papers
6	Setting	Conducted in the UK or an OECD Country Reporting an intervention that was conducted in a workplace	Reporting interventions in health sector workplaces (e.g., a hospital)
7	Outcome Measured	Quantitative data on workplace stress or anxiety (burnout, perceived stress, measures of cortisol levels, etc.)	Change in outcome level not reported
8	Population Group	Reporting an intervention which provides data on its effects on older workers in the workforce	Data reported with no desegregation by workers age or no evidence that included workers would be considered as “older”
9	Study Design	Experimental Designs Randomised Controlled Trials Non-Randomised Trials Before and After Studies	Qualitative papers (i.e., interview, focus group or ethnographic studies reporting experience of impressions)
10	Study Aim	Where at least one of the objectives of the intervention or programme is to reduce workplace stress	

**Table 5:** Results from the Risk of Bias Critical Appraisal.

Study (Author, Year)	Domain 1 (Randomisation Process)	Domain 2 (Deviations from intended Interventions)	Domain 3 (Missing Outcome Data)	Domain 4 (Measurement of Outcome)	Domain 5 (Selection of the Reported Results)	Domain 6 (Overall Bias)
Hughes, 2011 [17]	2	1	2	2	1	2
Malarkey, 2013 [18]	2	1	1	1	1	1
Aikens, 2014 [19]	2	2	1	1	1	1
Cook, 2015 [20]	2	1	2	2	1	2
Largo-Wight, 2017 [21]	2	1	1	2	2	2
Limm, 2011 [22]	2	1	1	2	1	1
Hoeve, 2021 [23]	1	1	1	1	1	1
Ojala, 2019 [24]	2	1	2	2	2	2
Fischetti, 2019 [25]	2	1	1	3	2	2
Calogiuri, 2016 [26]	2	1	2	2	2	2

Key for Table 5: 1 = Low Risk of Bias. 2 = Some Concerns. 3 = High Risk of Bias.

**Table 6:** Description of Methods and Findings.

**Study** **(Author, Year)**	Intervention Type	Duration	Location	Outcome Measurement Method	Data Collection Type	Findings	Intervention Deemed Effective? ^1^
Low risk of bias							
Malarkey, 2013 [18]	Psychological	8 Weeks	Office	Perceived Stress Scale Questionnaire	Self-report	No significant differences were seen at follow-up	No
				Cortisol Levels	Blood test & Saliva sample	No significant changes were noted	
Aikens, 2014 [19]	Psychological	7 Weeks	Online	Perceived Stress Scale Questionnaire	Self-report	23.1% decline in perceived stress	Yes
Limm, 2011 [22]	Psychological	8 Months	Office	Stress Reactivity Scale (SRS)	Self-report	The reduction in SR in intervention group (from 54.5 to 50.2) was significantly higher than in the control group (from 54.5 to 52.7)	Mixed
				Salivary Cortisol Levels	Saliva samples	No effect observed	
Hoeve, 2021 [23]	Psychological	6 Weeks	Office	Depression, anxiety, and stress scale (DASS)	Self-report	General stress score decreased from a group mean of 1.05 to 0.58	Yes
				Police Stress Questionnaire (PSQ-Op)	Self-report	Occupational Stress scores decreased from a group of 3.17 to 2.84	
Some bias concerns							
Hughes, 2011 [17]	Educational & Psychological	12 Months	Office	Perceived Stress Scale Questionnaire	Self-report	No quantitative data reported ^2^	No
Cook, 2015 [20]	Physical & Psychological	3 Months	Online	Symptoms of Distress Likert scale questionnaire	Self-report	No difference between groups	No
				Coping with Stress questionnaire	Self-report	No differences between groups on coping with stress	
Largo-Wight, 2017 [21]	Psychological	4 Weeks	Outside	Perceived Stress Scale Questionnaire	Self-report	Mean PSSQ score decreased from 62.3 to 61.2 in intervention group, compared to 66.2 to 64.2 decrease from control group	Yes
Ojala, 2019 [24]	Psychological	9 Months	Implied to be in Office	Bergen Burnout Inventory (BBI)	Self-report	Total BBI decreased from 36.9 to 33.9 in intervention group, at follow up	Yes
				Utrecht Work Engagement Scale (UWES)	Self-report	UWES increased from 4.3 to 4.5 in intervention group, at follow-up	
Fischetti, 2019 [25]	Physical & Education	8 Weeks	Office	Occupational Stress Indicator	Self-report	Scores for Job as a source of stress decreased from 30.7 to 25.2	Yes
				Short-Form 12 Questionnaire	Self-report	Increases in scores with significant changes from pre- to post-intervention (48.2 to 53.4)	
Calogiuri, 2016 [26]	Physical	2 Weeks	Outside	Physical Activity Affective Scale Questionnaire	Self-report	Higher ratings for PAAS, in relation to Positive Affect in the intervention group	Mixed
				Cortisol Awakening Response (CAR)	Blood Test	No significant differences between groups	

^1^ For the Intervention Deemed Effective Column: Yes = the paper’s authors classify the intervention as effectiveness and/or successful in the text of the paper. Mixed = the paper has unclear conclusions regarding the effective of the intervention report. No = the intervention was not deemed as being effective by the authors of the paper. ^2^ In Hughes et al.’s paper there are no quantitative data for stress levels in the main text. However, a supplementary paper with that data was said to be available via contact with the Journal or Author. After contacting both, no response was received.

## Data Availability

Not applicable.

## Supplementary Materials

The following supporting information can be downloaded at: https://www.mdpi.com/article/10.3390/ijerph19159202/s1, PRISMA 2020 Checklist.

## Appendix A

**Table A1:** Data Extraction Form from Primary Dataset.

Study Number	1	2	3	4	5
**Data Set Decision**	PRIMARY DATA SET	PRIMARY DATA SET	PRIMARY DATA SET	PRIMARY DATA SET	PRIMARY DATA SET
**Study Information**					
**Database No.**	WoS154	WoS321	O1587	O1320	O1544
**Author(s)**	Francesco Fischetti, Stefania Cataldi, Francesca Latino & Gianpiero Greco	Giovanna Calogiuri, Katinka Evensen, Andi Weydahl, Kim Andersson, Grete Patil, Camilla Ihlebæk and Ruth Raanaas	Royer F Cook, Rebekah K Hersch, Dana Schlossberg, Samantha L Leaf	William B. Malarkey, David Jarjoura, Maryanna Klatt	Susan L. Hughes, Rachel B. Seymour, Richard T. Campbell, James W. Shaw, Camille Fabiyi and Rosemary Sokas
**Year**	2019	2016	2015	2013	2011
**Link to Article**	https://rua.ua.es/dspace/bitstream/10045/96031/1/JHSE_14_Proc4_53.pdf	https://brage.inn.no/inn-xmlui/bitstream/handle/11250/2380639/Work_2015_Calogiuri.pdf?sequence=1&isAllowed=y	https://www.jmir.org/2015/3/e82	https://www.ncbi.nlm.nih.gov/pmc/articles/PMC3528077/	https://ajph.aphapublications.org/doi/full/10.2105/AJPH.2010.300082
**Access Date**	8 June 2022	8 June 2022	8 June 2022	8 June 2022	8 June 2022
**Title of Article**	“Effectiveness of multilateral training didactic method on physical and mental wellbeing in law enforcement”	Green exercise as a workplace intervention to reduce job stress. Results from a pilot study	A Web-Based Health Promotion Program for Older Workers:Randomized Controlled Trial	Workplace based mindfulness practice and inflammation: a randomized trial.	Comparison of two health-promotion programs for older workers.
**Country**	Italy	Norway	United States	United States	United States
**Intervention**					
**Study Aim**	to investigate the effects of an 8-week multilateral training program on physical and mental wellbeing in policemen	to explore possible effects of green-exercise interventions in the workplace on psychological and physiological indicators of stress.	to evaluate the impact of a multimedia Web-based health promotion program on central health attitudes and practices of older workers.	This study focused on working adults who could benefit from lifestyle intervention strategies. In comparing the mindfulness intervention to the lifestyle education program, we focused on three biologic measures of chronic stress and inflammation	to examine the effects of 2 worksite health-promotion interventions (compared with a health-education control) on older workers’ healthy behaviors and health outcomes.
**Type of Intervention**	Multilateral training program on physical and mental well-being	Exercise-based Intervention	Web-based program to address a wide variety of health behavior topics, including physical activity, healthy eating, stress management, and tobacco cessation	Mindfulness Intervention	health-promotion intervention and health-education control
**Duration of Intervention**	8 Weeks	2 Weeks	3 months	8 weeks	12 months
**Location of Intervention**	Police Offices	outdoors in a green/nature area or in an indoor exercise-setting	Online intervention	University Offices	at university worksite
**Study Design**	Randomised Controlled Trial	Between Subjects Randomised Controlled Trial	Randomised Controlled Trial	randomized controlled trial	randomized controlled trial
**Methods**					
**How were participants recruited?**	recruited in the police offices of the Puglia region (Italy) between January and February 2019, healthy males belonging to the State Police voluntarily participated	healthy employees, sedentary or moderately active, in two workplaces in a small town in the north of Norway who responded to an invitation to participate in the study by e-mail	A recruitment flyer briefly describing the purpose of the study was emailed by company officials to all employees 50 years of age and older (approximately 2500 employees) located in multiple US offices of a global information technology company. The flyer stated that the study was being conducted by a research organization through a grant from the National Institutes of Health. The flyer also explained that participants would receive US $25 for completing the first questionnaire and US $25 for completing the second questionnaire, and that their name would be entered into a drawing in which 1 participant would receive US $500 during each questionnaire round. Interested employees who fit the inclusion criteria (age 50 and older) were instructed to contact the project staff directly by email or telephone.	PARTICIPANTS: were recruited from faculty and staff of The Ohio State University. Advertising promoted the trial as a life style intervention program and the types of interventions were not specified, with participants unaware of the intervention type until the first day of the actual intervention	Participants were recruited via announcements on staff listservs, targeted e-mails, staffed recruitment tables at events in highly trafficked buildings, and flyers posted throughout the university.
**Occupation of Participants**	Police Officers	Office Workers	employees of a large global information technology company	University Faculty Staff	Research participants were older support and academic staff at the University of Illinois at Chicago
**Specific Focus on Older Workers?**	No	No	Yes	Not specific - but mean age is high	Yes
**Outcome Measurement**	the sources of stress and coping strategies, and the physical and mental state of health perceived were measured by the Occupational Stress Indicator and the Short Form-12	Self-reported affective state was measured by the Physical Activity Affective Scale (PAAS), which place feeling states within four factors corresponding to the quadrants circumplex model of affect and arousal: ‘Positive Affect’, ‘Tranquility’, ‘Negative Affect’, and ‘Fatigue’ AND Two measures of cortisol as an indicator of stress were used: Cortisol Awakening Response (CAR) and serum concentration. Saliva samples were selfadministered by the subjects for the determination of the CAR the morning after each session.	Twelve items assessing the type of strategies one uses to cope with difficult situations and events. Questions are answered on a 4-point scale ranging from 1 (never) to 4 (almost always); higher score = better coping. Typical questions included “I often put things aside for a while to get perspective on them” and “I decide certain problems are not worth worrying about”	CRP, IL-6, Cortisol, BP and Perceived Stress Scale Questionnaires	We investigated change from baseline to 6 months and 12 months in 4 measures of stress. We used the Perceived Stress Scale to measure overall stress during the preceding month. We used a 4-item scale developed by Lorig et al. to assess health-related stress, and we used the Brief COPE to assess use of positive and negative coping behaviors
**Data Collection Style**	Self report questionnaires	Self report questionnaires and Cortisol levels in serum were measured by professional nurses through blood test the morning after each session between 8:00 and 9:00 AM	self report survey	Via blood tests, saliva samples for cortisol and self reported questionnaires	self reported questionnaire
**How were the Results analysed?**	Pre and Post Intervention	Comparison to Control, and Pre and Post Intervention	Comparison to Control, and Pre and Post Intervention	Comparison to Control, and Pre and Post Intervention	Comparison to control and other intervention
**Results**					
**No. of Participants**	20	14	278	186	423
**Dropout Rate**	0 Dropouts	11: 3 dropouts/lost to f/u	Zero Dropouts	16 dropouts/lost to follow up	56 dropouts/lost to follow up
**Age of Workers**	Mean = 46.8 (SD 3.9)	Range = 41–57	Participants ranged in age from 50 to 68 years	Mean age = Education group—49, MBI-Id—51	Mean age = 51, participants were all aged 40 years and older
**Any other condition of workers?**	No	No	No	No—participants were excluded if they had a known condition which enhances inflammation and a psychiatric disorder other than depression	No
**Change in Stress Levels**	experimental group perceived less stress, showed a more realistic attitude towards the various working situations and a greater perception of physical and mental wellbeing than control group (*p* < 0.05).	Concerning the PAAS components, a significant effect of group was found for Positive Affect when corrected for baseline values, with higher ratings reported by the nature group No significant differences between groups were found for cortisol concentration in serum	There were no differences between the program and control groups on symptoms of distress or coping with stress. The estimated adjusted posttest different between program and control group is 0.01 for Coping with Stress	Cortisol at Baseline in Education group was 0.15, mean at follow up is 0.12; MBI-id group at baseline is 0.10 and followup mean is 0.11. The mindfulness intervention was effective as the MBI-ld group demonstrated greater self reported mindfulness than the education group at 2 months. This difference was sustained at 6 and 12 months: With measures of depression (CES-D), stress (Perceived Stress Scale), sleep quality (PSQI), no significant differences were found at 2-months. The global test across all five produced a p-value of 0.91. The PSS measure, however, asked the questions in reference to the past week rather than the past month as is the standard format, which permitted an evaluation of the intervention at completion.Using an extensive pre and post MBI-ld salivary cortisol sampling protocol we saw no decrease in cortisol levels	No significant differences were seen for COACH or RealAge participants on any of the 4 stress outcomes at either time point
**Intervention deemed effective/** **successful**	It has been found that police officers perceived fewer sources of pressure after intervention and specifically: working days are lived with less stress	Green-exercise at the workplace could be a profitable way to manage stress and induce restoration among employees.	Significant program effects were not shown on measures of stress	The trial was designed to test whether MBI-ld was superior to an education control in lowering cortisol, IL-6 and CRP immediately at the end of the interventions. We did not confirm this hypothesis. MBSR in smaller observational studies has been reported by some but not all investigators to lower cortisol levels when the intervention is compared to a wait list control group	The differences in the findings of our respective studies raises important questions about whether a dose---response relationship exists between comprehensiveness of services offered, and whether certain program components, such as incentives, are more effective and more critical than others.
**Key Conclusions**	public policies are needed to promote the practice of physical activities as continuing education, including leisure and sports activities, and to promote the psychological stability, work efficiency, changes in living habits, improvements in wellbeing and, therefore, improvements in quality of life.	it also provides some evidence that green-exercise interventions in the workplace can be a more valuable resource than ‘traditional’ indoor exercise in promoting health among employees, especially reducing psychological as well as physiological stress.	A Web-based health promotion program showed promise for making a significant contribution to the short-term dietary and exercise practices of older working adults. The findings suggest that a multimedia Web-based program could be a promising vehicle for delivering health promotion material to older working adults.	MBI-ld significantly enhanced mindfulness by 2 months and it was maintained for up to a year when compared to the education control. We did not see any significant changes in self-report measures for depressive symptoms, perceived stress, or sleep quality. Most but not all MBSR investigations have found improvements in these areas when the intervention is compared to a wait list control group. We have performed a randomized trial with a compressed MBSR intervention in which instruction and practice occurred in the workplace. This reduced the barriers commonly mentioned for non-participation in MBSR programs. Adherence to the program was greater than 90% for 8 weeks (evidenced by weekly attendance/practice sheets) even though the subjects were unaware that mindfulness meditation was one of the lifestyle interventions being offered. Additionally, mindfulness was achieved and sustained for at least one year. It is possible that a more intense intervention would have produced more significant effects. We conclude that MBI-ld should be more fully investigated as a low-cost self-directed complementary strategy for reducing inflammation.	If we can reach older adults while they are still working and engage them in sustained health-promotion activities, we may be able to delay morbidity onset, thereby reducing cost to employers as well as future Medicare expenditures
**Discussion**					
**Author Identified Weaknesses**	the small number of police officers recruited due to the difficulties encountered during the organizational phase in obtaining the necessary authorizations and having the subjects available. Moreover, the voluntary sample is not representative of the entire population of the law enforcement and therefore it is not possible to generalize the results. the results obtained could provide important indications for future studies aimed to know the effects of physical training with a multilateral approach on the occupational stress management	due to a small-sample size, the generalizability of the results is quite limited. Using a between-subjects design represented a further limitation. a within-subjects design was preferred as in previous studies it was shown that nature-based interventions can have long lasting effects on physiological parameters	the reliance on self-reports. because of the particular characteristics of the sample, caution should be exercised in generalizing these findings to workforces that are less educated and affluent	A limitation of our study was omission of wait list control group as the impact of the MBI-ld may have achieved significance in comparison, indicating the. potential impact of such a workplace intervention.	the interventions were tested with staff at an inner-city university who may have had higher levels of education than do workers in other industries. Thus, the generalizability of the findings to workers in other settings requires further testing.

**Table A2:** Data Extraction Form from Secondary Dataset.

Study Number	6	7	8	9	10
**Data Set Decision**	SECONDARY DATASET	SECONDARY DATASET	SECONDARY DATASET	SECONDARY DATASET	SECONDARY DATASET
**Study Information**					
**Database Number**	WoS62	P144	O1594	O1575	O1130
**Author(s)**	Machteld Hoeve, Esther L. de Bruin, Floor van Rooij and Susan Bögels	Erin Largo-Wight, Peter S. Wlyudka, Julie W. Merten & Elizabeth A. Cuvelier	Birgitta Ojala, Clas-Håkan Nygård, Heini Huhtala, Philip Bohle and Seppo T. Nikkari	Heribert Limm, Harald Gündel, Mechthild Heinmüller, Birgitt Marten-Mittag, Urs M Nater, Johannes Siegrist, Peter Angerer	Kimberly A. Aikens, John Astin, Kenneth R. Pelletier, Kristin Levanovich, Catherine M. Baase, Yeo Yung Park, and Catherine M. Bodnar
**Year**	2021	2017	2019	2011	2014
**Link to Article**	https://link.springer.com/content/pdf/10.1007/s12671-021-01631-7.pdf	https://www.tandfonline.com/doi/pdf/10.1080/15555240.2017.1335211?needAccess=true	https://www.mdpi.com/1660-4601/16/1/80	https://oem.bmj.com/content/68/2/126?casa_token=4CAKgnx0r2wAAAAA%3Acw336eYaWoATNiEpIkpbAJuA7PAdti_Std6nW0lXmrjtAAudRItJdD0TEBaY6Rw8eeCpwbmjVvTs	http://affinityhealthhub.co.uk/d/attachments/mindfulness-goes-to-work-impact-of-an-online-intervention-1498490157.pdf
**Access Date**	8-June-2022	8-June-2022	8-June-2022	8-June-2022	8-June-2022
**Title of Article**	Effects of a Mindfulness-Based Intervention for Police Officers	Effectiveness and feasibility of a 10-minute employee stress intervention: Outdoor Booster Break	A Cognitive Behavioural Intervention Programme to Improve Psychological Well-Being	Stress management interventions in the workplace improve stress reactivity: a randomised controlled trial.	Mindfulness goes to work: impact of an online workplace intervention.
**Country**	Netherlands	United States	Finland	Germany	Michigan, United States
**Intervention**					
**Study Aim**	to increase knowledge on the effects of a mindfulnessbased intervention in police officers and potential mechanisms of change by relating changes in facets of mindful awareness to changes in stress	to explore the feasibility and efficacy of a brief work break outside (Outdoor Booster Break) among office employees.	to evaluate a cognitive behavioural intervention as an early rehabilitation strategy to improve employees’ well-being	to test the long-term effect of this SMI on acute perceived reactions to stress at work (stress reactivity) after 1 year, as the primary endpoint	to determine whether a mindfulness program, created for the workplace, was both practical and efficacious in decreasing employee stress while enhancing resiliency and well-being.
**Type of Intervention**	Mindfulness-based Intervention	Outdoor Booster Break environmental intervention	Cognitive Behavioural Intervention Programme	stress management intervention	online mindfulness workplace intervention
**Duration of Intervention**	6 Weeks	4 weeks	9 months	8 months	7 week
**Location of Intervention**	Police offices	University office	Not Specified—facilitated by an interdisciplinary, goal-oriented multi professional team (a doctor, an occupational physiotherapist, an occupational psychologist, and a nurse)	at worksite	Online (at work)
**Study Design**	Quasi Experimental Study Design	Randomised Controlled Trial	Non Randomised Controlled Trial	randomized controlled trial	randomized controlled study
**Methods**					
**How were participants recruited?**	Participants were recruited by the Dutch trade union. Before the training, during a company away day, members of the trade union had the opportunity to participate in a mindfulness 2-hour workshop, led by SB and one of the mindfulness trainers, in order to inform them about the possibility of participating in a mindfulness training.	A census of university office staff in the southeast in springtime was invited to participate	Participants were volunteers who met the inclusion criteria for the study: Being employed in the public sector and working as permanent or long-term temporary staff with at least one year of service.	All lower and middle level managers (n¼262), each responsible for a specific unit within production and for the management of 50 workers, on average, were eligible. All participants were invited to a 1.5 h medical and psychological examination by an experienced team consisting of a psychologist (HL) and a physician (MH). Written informed consent was obtained. All volunteers were required to complete a battery of questionnaires, participate in a basic physical examination with blood sampling and collect saliva samples the next working day. This initial health check included feedback to each participant a few days later.	Participants were drawn from a sample of 600 Dow employees, located in Midland, Michigan, who had completed a health risk assessment (comprehensive questionnaire and biometrics) in the preceding 6 months. All employees are invited for health risk assessment with employees in given departments being scheduled throughout the year. This recruitment allowed for study access to a good cross section of employees because the standard process of invitations would include all elements of the employee base. Study participant recruitment occurred from March to April 2012 and consisted of one e-mail notification, which described the free mindfulness-based stress management program. The e-mail notification explained that the purpose of the program was to help employees reduce and manage workplace stress.
**Occupation of Participants**	Police Officers	University office staff	Public sector workers	lower and middle level managers at an international manufacturing plant located in Southern Germany	General employees at a chemical company
**Specific Focus on Older Workers?**	No	No	No	No	No
**Outcome Measurement**	Symptoms of stress were measured by four instruments that measure different types of stress, including general feelings of stress and tension, physical stress, occupational stress during police work, and stress symptoms that are related to a traumatic event (i.e., PTSD symptoms).	Perceived stress was measured two times–at pretest and posttest for both conditions with a self-report perceived stress instrument. The Perceived Stress Questionnaire (PSQ)	The measurement tools used in this study were the Bergen Burnout Inventory (BBI) and the Utrecht Work Engagement Scale (UWES).BBI 15 was used to measure burnout. It includes three sub-dimensions: Exhaustion (five items), cynicism (five items), and sense of inadequacy (five items). UWES 9 was used to define three dimensions of work engagement: Vigour (three items), dedication (three items), and absorption (three items)	Self-reported stress reactivity was measured with the 29-item Stress Reactivity Scale (SRS). Biological stress indices were measured using levels of salivary cortisol as an indicator of hypothalamic-pituitary-adrenal axis activity, and salivary a-amylase, reflecting basal activity of the sympathetic nervous system	The Perceived Stress Scale (PSS-14) was used to assess participants’ levels of psychological stress. The PSS-14 is a well-validated stress measurement tool whose items are designed to tap into how unpredictable, uncontrollable, and overloaded individuals find their lives
**Data Collection Style**	Self report questionnaires	self-reported perceived stress 4-point Likerttype scale from participants	self reported questionnarie	self reported questionnaire and saliva samples	self reported questionnaire
**How were the Results analysed?**	Pre and Post Intervention, plus Follow Up	Comparison to control group, and pre and post intervention	Comparison to Control, and Pre and Post Intervention	Comparison to Control, and Pre and Post Intervention	Comparison to Control, and Pre and Post Intervention
**Results**					
**No. of Participants**	82	37 (Treatment had Outdoor Break, Control had Indoor Break)	779	174	89
**Dropout Rate**	19 dropouts	No Dropout Mentioned	217 dropouts	20 dropouts/lost to follow up	23 dropouts/failed to follow up
**Age of Workers**	30–63 (mean = 49)	Average age of 48.8	mean age of subjects was 49.9 years (range 21–64 years)	Mean age = 40.9. Aged 18–65 years with more than 2 years left before retirement	18-65
**Any other condition of workers?**	No	No	No	No	No
**Change in Stress Levels**	Improvement in mindful awareness (mindfulness total scale) was marginally significantly associated with a reduction in general feelings of stress and tension. Further, an increase in attention was significantly associated with reductions in general stress and occupational stress.	Observed average posttest stress scores were lower for both the control group and the treatment group (Mean PSQ Stress, Control Pre = 66.25, Control Post 64.25, Treatment Group Pre 65.25, Treatment Group Post 61.25). Posttest stress was 4.22 points lower (95% CI: or the treatment group compared to controls	Total BBI 15 values for the intervention group were 36.9 (standard deviation (SD) 11.8) at baseline and 33.9 (SD 12.3) at follow-up. The change from baseline was −3.0 (*p* < 0.001). Values for the control group were 37.6 (SD 12.2) at baseline and 37.5 (SD 14.4) at follow-up. The change from baseline was 0.1 (*p* = 0.912). The difference in changes between groups was statistically significant (*p* = 0.023).	The reduction in perceived stress reactivity in the intervention group (from 54.5 to 50.2) was significantly higher than in the control group (from 54.5 to 52.7). For cortisol, no effect of the intervention was observed. Self-perceived stress reactivity assesses typical cognitive, emotional and physiological reactions to different stressful situations. High stress reactivity scores have been shown to significantly correlate with a variety of other psychological measures of distress such as depression or anxiety.	perceived stress declined by 23.1% from baseline values in the follow up 6 months. Participants also reported a decline in weekly high stress episodes by 33%, which is a significant downward trend (*p* < 0.001).
**Intervention deemed effective/successful**	Police officers significantly benefited from the mindfulnessbased intervention	taking a work break in general resulted in a reduction of stress among the employees. All employees benefited from a reduction of generalized stress after 4 weeks of daily work breaks. But, as expected, the participants randomized into the Outdoor Booster Breaks resulted in significantly greater reduction in stress over the 4-week study than the participants who took a standard indoor work break.	The principal finding of this study is a statistically significant improvement in several measures of psychosocial well-being (BBI 15, UWES, stress, depression) for participants who completed the cognitive behavioural intervention programme.	Our approach proved to be feasible in the workplace setting, it was well accepted and it produced selected favourable behavioural and physiological effects	The present findings have significant potential implications for corporate health and human performance. The program studied was a mindfulness intervention, which was modified in length, content, and messaging to fit workplace needs and delivered through an on-line platform that included personal coaching. Overall, the ESs obtained in this study were in the moderate to large range and were either maintained, or further improved, over time. This indicates that a shortened, Web-based mindfulness program can replicate the results of traditionally delivered MBSR.
**Key Conclusions**	Mindfulness-based intervention appears beneficial for police officers. Further, increases in both attention and acceptance skills such as acting with awareness and non-judging seem to be most important in explaining reductions of stress in police officers.	This study points to several implications for employers and worksite health promoters. First, environmental stress interventions such as Outdoor Booster Breaks are relatively simple to implement. Creating healthy workplaces is a health promotion effort that may be more practical and feasible than many other labor-intensive health promotion efforts. Creating a healthful work environment—with the purposeful use of nature contact—is a simple and practical step to improve employee health and productivity. Environmental improvements require little, if any, commitment and effort from the employee. In a way, improving the workplace environment to foster health among employees is one way to “set the employees up for success.”	This study suggests that a cognitive behavioural intervention achieved significant improvements in several measures of mental health. The results imply that this kind of intervention is needed to give early support on mental health issues for the working-age population.	SMI based on work stress theory, is effective in reducing perceived stress reactivity and sympathetic activation in lower and middle management employees. Other mental health parameters and ERI show a tendency towards improvement. These beneficial effects are present 1 year later.	This on-line mindfulness intervention seems to be both practical and effective in decreasing employee stress, while improving resiliency, vigor, and work engagement, thereby enhancing overall employee well-being.
**Discussion**					
**Author Identified Weaknesses**	we examined the effectiveness of an adapted corporate mindfulness training. Although adaptations to the work situation of police officers were made, such as in the enquiries and discussion after the meditations and the examples being used in exercises, no cultural adaptations were made to the content of the training, which could have diminished the positive effects. Second, findings might not be generalizable to the entire population of police officers. Although our sample size is larger than most earlier studies on the effects of mindfulness-based intervention in police officers, the number of participants was small due to attrition at post-test and follow-up. senior workers are generally overrepresented in unions in the Netherlands. Therefore, it should be kept in mind that the results may apply only to more experienced police officers and less to relatively young or inexperienced officers.	the sample size and group sizes were small. the generalizability of the findings to male workers. Future researchers should assess the impact of Outdoor Booster Breaks with greater male representation. Also perceived stress was the only outcome measured.	One limitation is that the participants represent a relatively small population in Finland. The intervention and control groups were selected partly according to the participants’ own interests. Question-based research may suffer from bias if the participants feel satisfied with the service and therefore respond positively when they answer the second time.	the effects were only moderate and health effects still have to be demonstrated in longer follow-up studies. This also points to the fact that improving working conditions must remain a primary goal of stress prevention even though this is sometimes hard to attain in practice	this study had results from a relatively small number of participants (*n* = 79), creating the need for a larger randomized control trial to confirm the results. In addition, 12-month follow-up was not completed to avoid overburdening busy employees. This study limitation precludes us from making a more definitive assessment regarding the long-term effectiveness of the mindfulness intervention
